# AI-powered drug discovery for neglected diseases: accelerating public health solutions in the developing world

**DOI:** 10.7189/jogh.15.03002

**Published:** 2025-01-10

**Authors:** MD Nahid Hassan Nishan

**Affiliations:** Department of Public Health, North South University, Dhaka, Bangladesh

## Abstract

The emergence of artificial intelligence (AI) in drug discovery represents a transformative development in addressing neglected diseases, particularly in the context of the developing world. Neglected diseases, often overlooked by traditional pharmaceutical research due to limited commercial profitability, pose significant public health challenges in low- and middle-income countries. AI-powered drug discovery offers a promising solution by accelerating the identification of potential drug candidates, optimising the drug development process, and reducing the time and cost associated with bringing new treatments to market. However, while AI shows promise, many of its applications are still in their early stages and require human validation to ensure the accuracy and reliability of predictions. Additionally, AI models are limited by the availability of high-quality data, which is often sparse in regions where neglected diseases are most prevalent. This viewpoint explores the application of AI in drug discovery for neglected diseases, examining its current impact, related ethical considerations, and the broader implications for public health in the developing world. It also highlights the challenges and opportunities presented by AI in this context, emphasising the need for ongoing research, ethical oversight, and collaboration between public health stakeholders to fully realise its potential in transforming global health outcomes.

Neglected diseases, including malaria, tuberculosis, leishmaniasis, and Chagas disease, disproportionately affect populations in low- and middle-income countries (LMICs). These diseases continue to pose a significant burden on public health, with the World Health Organization (WHO) estimating that more than one billion people are affected by neglected tropical diseases globally [1]. Traditional drug discovery processes, driven largely by commercial interests, have often overlooked these diseases due to a lack of financial incentives, resulting in a significant gap in the availability of effective treatments for these diseases, exacerbating health disparities between high- and low-income regions [2].

Artificial intelligence (AI) in drug discovery offers a novel approach to addressing these challenges. With its capacity to analyse vast data sets and identify patterns that are difficult for humans to detect, AI has the potential to enhance drug discovery processes [3]. Recent advancements in AI have significantly identified potential therapeutic targets, including those for malaria and tuberculosis. In malaria research, for example, machine learning (ML) algorithms have been employed to screen large chemical libraries, identifying novel drug candidates with antimalarial properties. One such study led to the identification of several promising compounds, which are now in preclinical evaluation [4]. Similarly, AI applications in tuberculosis research have been pivotal in identifying drug candidates effective against drug-resistant strains of *Mycobacterium tuberculosis*. By analysing vast data sets, AI models have accelerated the discovery of new therapeutic options for this challenging disease [5].

In addition to these advancements, AI has played a crucial role in drug repurposing for neglected diseases. For example, the antiarrhythmic drug amiodarone was identified as a potential treatment for Chagas disease through AI, showcasing its ability to find new uses for existing medications [6]. Likewise, AI-assisted models have identified existing drugs with potential antileishmanial activity, offering a fast-tracked route to clinical application [7]. These examples illustrate the growing potential of AI in accelerating drug discovery processes, particularly for diseases that have long been overlooked by traditional pharmaceutical research.

However, it is important to note that AI alone cannot ‘transform’ drug discovery; these models require human oversight to ensure that their predictions are accurate and relevant to clinical practice. Many AI applications in drug discovery are still in the early stages of development and must undergo rigorous testing and validation before their findings can be applied in real-world settings. By leveraging AI, however, researchers can accelerate the identification and development of new drugs for neglected diseases, potentially transforming public health outcomes in the developing world [8]. Yet for these transformative outcomes to be realised, it is essential to address the broader systemic challenges such as weak health care infrastructure, limited access to medications, and socio-political barriers. AI-driven solutions must be integrated into comprehensive health system strengthening initiatives that include building local capacity, improving supply chains, and ensuring equitable access to innovative treatments [9]. While AI holds promise in predicting drug efficacy, optimising drug candidates, and streamlining the drug development process, its success is heavily dependent on the availability of high-quality data and the integration of human expertise throughout the development pipeline [10].

AI technologies, including ML, deep learning, and natural language processing (NLP), are being increasingly integrated into drug discovery. These technologies allow for the analysis of complex biological data, including genetic information, chemical structures, and disease models, to identify potential drug candidates with greater precision and speed than traditional methods [10]. Their use in drug repurposing – identifying new uses for existing drugs – is particularly promising for neglected diseases, reducing the time and cost associated with drug development [11].

However, integrating AI into drug discovery for neglected diseases presents a number of challenges. Data availability and quality are primary concerns, as AI algorithms require large, high-quality data sets to be effective. In the context of neglected diseases, data may be sparse or of lower quality, particularly in regions where these diseases are most prevalent [8]. Furthermore, ethical considerations related to data privacy, algorithmic bias, and the potential for AI to exacerbate existing health disparities must be addressed to ensure AI-powered drug discovery benefits are realised equitably.

Therefore, this viewpoint explores the current state of AI-powered drug discovery for neglected diseases, focussing on its applications, ethical considerations, and implications for public health in resource-limited settings. By examining AI's potential and challenges, it aims to highlight its transformative impact on global health, particularly for populations historically underserved by the pharmaceutical industry.

## TECHNOLOGICAL BACKGROUND

AI, particularly ML and deep learning, has emerged as a powerful tool in drug discovery. These technologies enable the rapid analysis of vast and complex data sets, essential for identifying potential drug candidates. In the context of neglected diseases, AI can efficiently screen thousands or millions of compounds to identify those with therapeutic potential [12].

For example, ML algorithms are designed to recognise patterns in data and make predictions based on those patterns. In drug discovery, ML can predict the biological activity of chemical compounds, identify potential drug targets, and optimise drug candidates [13]. However, one of the limitations of ML models is their reliance on data quality. As a consequence, poor, incomplete, or noisy data sets can significantly reduce their accuracy. Additionally, when dealing with small or unbalanced data sets, which is often the case for neglected diseases, ML models may struggle to produce reliable predictions [14].

Deep learning, a subset of ML, involves neural networks with multiple layers to model complex data relationships. It has been particularly successful in drug discovery, as it can automatically extract features from raw data, such as chemical structures, to make predictions about drug efficacy [15]. A notable application of deep learning in drug discovery is the use of convolutional neural networks (CNNs) to analyse images of chemical compounds and predict their activity against disease targets. This approach has identified new drug candidates for diseases such as malaria and tuberculosis [16]. However, deep learning models, including CNNs, have limitations. They typically require large, high-quality data sets to perform effectively, which presents a challenge in the context of neglected diseases, where data are often sparse or incomplete. This can lead to issues such as overfitting, where the model performs well on training data, but struggles to generalise to new, unseen data [17]. Additionally, the data infrastructure needed to collect and maintain such data sets is often lacking in low-resource settings, further limiting the effectiveness of these AI technologies.

Another critical AI technology in drug discovery is NLP, which analyses and extracts information from unstructured text data, such as scientific literature, patents, and clinical trial reports. In the context of neglected diseases, this approach can identify potential drug candidates by analysing existing research and identifying gaps in the literature that may represent opportunities for drug development [18]. For example, NLP algorithms can mine scientific literature for mentions of specific chemical compounds, disease targets, or therapeutic strategies, and then prioritise those findings for further investigation. However, NLP also faces challenges, particularly when applied to neglected diseases where the research literature may be limited, outdated, or incomplete. This can result in missed information or incomplete data extraction, limiting the effectiveness of such algorithms in identifying viable drug candidates [19].

AI's role in drug repurposing – finding new uses for existing drugs – also holds significant promise. For example, AI algorithms can analyse data on existing drugs, including their chemical structures, mechanisms of action, and clinical trial results, to identify those that may be effective against neglected diseases. This approach has led to the identification of several promising drug candidates for diseases such as Chagas disease and leishmaniasis, which have historically received limited attention from the pharmaceutical industry [20].

## FOUNDATIONAL INSIGHTS

This viewpoint draws on a review of recent and relevant literature to provide insights into AI-powered drug discovery for neglected diseases. We conducted a search in PubMed, IEEE Xplore, and Google Scholar using relevant keywords such as ‘artificial intelligence’, drug discovery,’ ‘neglected diseases’, ‘machine learning’, and ‘developing world’. The inclusion criteria focussed on peer-reviewed, English-language quantitative or qualitative studies and conference papers published in the last two decades that offered empirical data, theoretical insights, or ethical discussions directly relevant to the application of AI in drug discovery for neglected diseases. Non-peer-reviewed literature and opinion pieces without empirical backing were excluded from this analysis.

Studies were critically evaluated based on their methodological rigour, relevance to the topic, and the strength of the evidence provided. To ensure a thorough evaluation, each study was assessed for its contribution to key themes such as technological advancements, practical applications, and ethical considerations. A narrative synthesis approach was employed to integrate the findings from the selected studies, allowing for the presentation of a cohesive and well-rounded perspective on the potential and challenges of AI in accelerating drug discovery for neglected diseases.

## APPLICATIONS AND IMPACT

The application of AI in drug discovery for neglected diseases has significantly accelerated the identification and development of new treatments, with the most notable application case being the use of ML algorithms to identify potential drug candidates for malaria. This disease remains one of the most significant public health challenges in the developing world, with hundreds of thousands of deaths each year, primarily in sub-Saharan Africa [21]. AI has been used to screen large libraries of chemical compounds to identify those with potential antimalarial activity. For example, researchers have used ML models to analyse the chemical structures of known antimalarial drugs and predict which other compounds might exhibit similar activity. This approach has led to the discovery of several novel drug candidates, some of which are now in preclinical or clinical development [22,23].

AI has also been applied to discovering new treatments for tuberculosis, another major public health threat in the developing world. Tuberculosis, particularly in its drug-resistant forms, poses a significant challenge to global health. AI has been used to identify compounds that can target drug-resistant strains of *Mycobacterium tuberculosis*, the bacteria responsible for the disease. In one example, ML models have predicted the efficacy of new drug combinations, optimised treatment regimens, and reduced the likelihood of resistance development [24]. This application of AI is crucial in addressing the growing challenge of multidrug-resistant tuberculosis, which poses a significant threat to global public health.

In addition to malaria and tuberculosis, AI has been instrumental in identifying drugs for other neglected diseases, such as Chagas disease, leishmaniasis, and schistosomiasis. A particularly effective application has been AI-driven drug repurposing, which identifies new uses for existing medications to combat these diseases [25]. Here, AI models analyse extensive data sets which include chemical structures, mechanisms of action, and clinical trial data of known drugs in order to uncover new potential applications [10]. For instance, AI identified amiodarone, an antiarrhythmic drug, as a potential treatment for Chagas disease. Similarly, AI-assisted models have successfully identified drugs with potential antileishmanial activity by recognizing patterns in chemical properties that align with known treatments for leishmaniasis [26]. By leveraging AI to identify new uses for existing drugs, researchers can bypass many of the costly and time-consuming steps in traditional drug development, bringing new treatments to market more quickly [27].

AI's impact on drug discovery extends beyond identifying new drug candidates; it is also being used to optimise the drug development process itself. For example, AI can design more efficient clinical trials by predicting patient responses to different treatments, optimising dosing regimens, and identifying biomarkers that can monitor treatment efficacy [28]. These advancements could significantly reduce the time and cost associated with bringing new treatments to market, making developing drugs for neglected diseases more feasible.

The scalability of AI-powered drug discovery is another critical advantage in the context of neglected diseases. Traditional drug discovery processes are often limited by the availability of resources, including funding, laboratory space, and human expertise. In contrast, AI algorithms can be scaled to analyse vast amounts of data and identify potential drug candidates across a wide range of diseases [29]. While this scalability is particularly important for neglected diseases, where traditional research efforts have often been constrained by limited resources, achieving it is not without challenges. Many developing countries face significant barriers, including the need for extensive computational resources and technological infrastructure, which are often limited or lacking. There is also a shortage of skilled personnel trained in AI and data science necessary to implement these technologies effectively. The financial investments required to establish and maintain the necessary infrastructure can also be prohibitive.

AI's integration into the drug development pipeline spans key stages, enhancing efficiency and effectiveness [30]. During preclinical testing, AI can predict the biological activity and toxicity of drug candidates, helping prioritise promising compounds. In clinical trials, AI optimises designs by predicting patient responses and enabling real-time data analysis, potentially expediting the approval process. By enabling more efficient use of available resources, AI can accelerate the development of new treatments for a broad range of neglected diseases.

## ETHICAL CONSIDERATION AND CHALLENGES

While the potential benefits of AI-powered drug discovery are substantial, significant ethical considerations must be addressed to ensure the responsible use of these technologies. One of the primary concerns is bias in AI algorithms. Specifically, AI systems are only as good as the data on which they are trained, and if these data sets are biased or incomplete, the resulting models may produce skewed outcomes. This is particularly concerning in the context of neglected diseases, where data may be limited or not fully representative of the populations most affected by these diseases [31]. For example, if an AI model is trained primarily on data from high-income countries, it may not perform as well when applied to populations in LMICs, where the burden of neglected diseases is highest. This bias can exacerbate existing health disparities and limit the accessibility of AI-driven treatments in underrepresented regions To mitigate this risk, it is essential to ensure that AI models are trained on diverse and representative data sets and that their predictions are validated across different populations and settings.

In addition to bias, the social justice implications of AI in drug discovery must be carefully considered. AI-driven innovation could inadvertently prioritise treatments for diseases prevalent in wealthier countries, while neglecting the needs of LMICs. The lack of affordable access to AI-based technologies in these regions can widen the gap in healthcare access. To address this, AI tools should be developed in a way that prioritises inclusivity, ensuring equitable benefits for all populations, particularly those most vulnerable to neglected diseases.

Another significant challenge in applying AI to drug discovery for neglected diseases is the limited availability and quality of data in LMICs. AI models rely heavily on large, high-quality data sets to generate accurate predictions [32]. However, in regions where neglected diseases are most prevalent, such as sub-Saharan Africa and parts of Southeast Asia, data on disease prevalence, clinical outcomes, and treatment efficacy may be sparse or of low quality. These data gaps hinder the effectiveness of AI models in identifying potential drug candidates.

Several strategies can be employed to address these issues. One approach is to develop collaborative data-sharing platforms that pool data from various sources, including research institutes, governments, and non-profit organisations. These platforms can help aggregate data sets from underrepresented regions, ensuring that AI models are trained on more diverse and comprehensive data [33]. Another solution is the use of synthetic data generation techniques, which simulate patient data or disease progression to supplement existing data sets. This method can enhance the predictive accuracy of AI models for neglected diseases, even when real-world data are limited [34]. Furthermore, investments in data collection infrastructure in LMICs, such as electronic health records and disease registries, are crucial for ensuring that high-quality data are consistently collected and standardised [35]. Federated learning is another promising strategy that allows AI models to be trained on decentralised data, enabling local data sets to remain within their country of origin while contributing to global research efforts [36]. This approach also helps address privacy concerns, while making localised data usable without the need for centralisation.

Another ethical consideration is the potential for AI-driven drug discovery to exacerbate existing health disparities. While AI has the potential to accelerate the development of new treatments for neglected diseases, it also requires significant computational resources and technical expertise, which may be lacking in LMICs [37]. This could lead to a situation where the benefits of AI-powered drug discovery are concentrated in wealthier nations, while the developing world continues to struggle with inadequate access to new treatments. Addressing this challenge will require concerted efforts to build capacity in AI and data science in low-resource settings and to ensure that the benefits of AI-driven drug discovery are equitably distributed. For example, partnerships between high-income and low-income countries could be established to share knowledge, resources, and expertise in AI and drug discovery.

Privacy and data security are also critical ethical issues in the use of AI for drug discovery. The collection and analysis of biological and health data are central to AI-powered drug discovery, raising concerns about how this data are stored, shared, and protected. Ensuring the privacy and security of sensitive health data are essential to maintaining public trust and preventing misuse [38]. This is particularly important in the context of neglected diseases, where vulnerable populations may be at greater risk of exploitation or discrimination. Transparent and accountable AI governance frameworks must be implemented to ensure that ethical principles are upheld throughout the drug discovery process. To address these concerns, it is crucial to implement robust data protection measures, including encryption, anonymisation, and strict access controls, to safeguard patient data.

Another ethical challenge is the potential for AI to reduce the role of human judgment in drug discovery. While AI can analyse data and make predictions with remarkable speed and accuracy, it lacks the nuanced understanding and ethical reasoning that human researchers bring to the drug discovery process. There is a risk that over-reliance on AI could lead to decisions that are not fully informed by ethical considerations or fail to consider the broader social and cultural context in which drugs will be used [38]. This raises the concern that over-reliance on AI could lead to decisions that fail to consider the broader social and cultural context in which drugs will be used. To mitigate this risk, it is essential to ensure that AI is used as a tool to support rather than replace human decision-making in drug discovery. Human oversight should be maintained throughout the drug development process to ensure that decisions are made in patients' best interests and that ethical considerations are fully addressed.

## REGULATORY AND LEGAL FRAMEWORKS

The integration of AI into drug discovery presents unique regulatory and legal challenges, particularly in LMICs, where such frameworks may not be fully equipped to handle the complexities of AI-driven innovations. Existing regulations for drug approval are often designed for traditional pharmaceutical development processes, which may not account for the use of AI algorithms in identifying and validating drug candidates.

One of the key challenges in this sense is ensuring that AI-powered tools meet the regulatory standards for safety, efficacy, and transparency. AI models must undergo rigorous validation to ensure that their predictions are reliable and reproducible, particularly when used to inform decisions about drug development and approval [39]. However, regulatory agencies in LMICs may lack the resources and technical expertise to evaluate AI-based approaches, posing a barrier to the widespread adoption of these technologies. To address these challenges, regulatory frameworks need to evolve to accommodate the use of AI in drug discovery. This could involve the creation of new guidelines for the evaluation and approval of AI-driven drug candidates, emphasising the need for transparency in how AI models make decisions and the requirement for human oversight throughout the drug development process. Capacity-building initiatives could be established to strengthen regulatory bodies in LMICs, ensuring they have the necessary tools and expertise to assess AI-driven innovations.

International cooperation will be crucial in facilitating the regulatory evolution needed for AI-driven drug discovery. Collaborative efforts between high-income and low-income countries could help harmonise regulatory standards, allowing for faster and more efficient drug approval processes. Organisations like the WHO and the International Council for Harmonization of Technical Requirements for Pharmaceuticals for Human Use could play a pivotal role in developing global regulatory standards for AI applications in drug discovery [40]. Potential solutions include the establishment of regulatory sandboxes, where AI-driven technologies can be tested in a controlled environment before full-scale implementation. These frameworks would allow regulatory bodies to assess the safety and efficacy of AI-powered drug discovery tools while still providing flexibility for innovation. Additionally, adopting fast-track approval processes for AI-discovered drugs, similar to existing pathways for orphan drugs or treatments for rare diseases, could expedite access to life-saving medications in LMICs.

## WAY FORWARD

The integration of AI into drug discovery for neglected diseases represents a significant advancement in the fight against some of the world’s most persistent public health challenges. The ability of AI to analyse vast data sets, predict drug efficacy, and identify novel drug candidates offers a promising solution to the long-standing issue of neglected diseases, particularly in the developing world. AI-powered drug discovery has already shown success in identifying new treatments for diseases like malaria and tuberculosis, demonstrating its potential to accelerate the development of effective therapies where they are needed most.

One of the key strengths of AI in drug discovery is its ability to rapidly screen large libraries of compounds and identify those with therapeutic potential. This capability is particularly valuable for neglected diseases, where there is often a lack of existing research and data on potential drug candidates [8]. By leveraging AI to analyse chemical structures, biological data, and disease models, researchers can identify promising drug candidates more quickly and precisely than traditional methods. This has already led to the discovery of several novel drug candidates for diseases like malaria and tuberculosis, which are now in various stages of development.

In addition to identifying new drug candidates, AI is also being used to optimise the drug development process itself. These advancements could significantly reduce the time and cost associated with bringing new treatments to market, making developing drugs for neglected diseases more feasible. This is particularly important in the context of neglected diseases, where traditional drug development efforts have often been constrained by limited resources and funding [41]. Despite its potential, AI in drug discovery requires human validation to ensure accurate and reliable predictions, as many applications are still in the early development stages. Additionally, AI's success is dependent on high-quality data, which is often limited for neglected diseases, making improved data collection in these regions essential.

However, the successful application of AI in drug discovery for neglected diseases is not without challenges. One of the primary concerns is the issue of data quality and availability. For AI algorithms to be effective, they require large, high-quality data sets. In the context of neglected diseases, data may be sparse or of lower quality, particularly in regions where these diseases are most prevalent [28]. This presents a significant challenge for AI-powered drug discovery, as the success of these algorithms heavily depends on the quality and representativeness of the data on which they are trained. To address this challenge, it is essential to invest in the collection and curation of high-quality data on neglected diseases, particularly in LMICs, where these diseases are most prevalent.

Another challenge is the ethical considerations associated with using AI in drug discovery. Issues such as data bias, privacy, and the potential for AI to exacerbate existing health disparities must be carefully addressed to ensure that the benefits of AI-powered drug discovery are realised equitably and responsibly [42]. To mitigate bias in AI models, particularly when working with data from underrepresented regions, federated learning offers a practical solution by allowing decentralised data to contribute to AI training without compromising privacy [43]. This ensures that data from LMICs can be included, improving model accuracy across diverse populations. Data privacy is further protected by keeping sensitive health data localised, with additional safeguards like data encryption and anonymisation. Governments, non-governmental organisations (NGOs), and academic institutions play pivotal roles in advancing AI-driven drug discovery in LMICs. Governments can, for example, create ethical AI policies and promote public-private partnerships to support underserved populations. NGOs are essential for capacity-building, offering training, and establishing data-sharing platforms to ensure equitable access to AI technologies [35]. Academic institutions, in turn, contribute by fostering innovation, building AI infrastructure, and collaborating on international research projects.

Global partnerships, such as those by the Global Health Innovative Technology Fund and the WHO, help tailor AI solutions to the unique challenges of LMICs, ensuring equitable benefits for all populations. Building AI capacity in LMICs requires concerted efforts in education, infrastructure, and collaboration. First, establishing AI and data science programmes in local universities can equip researchers with the necessary technical skills. Second, partnerships with international institutions can facilitate knowledge transfer and provide access to resources. Third, improving technological infrastructure, such as cloud computing and data storage facilities, is crucial to support AI research. Finally, fostering public-private collaborations will allow for the exchange of expertise and help fund AI projects that target neglected diseases. By implementing these capacity-building strategies, LMICs can develop a sustainable foundation for leveraging AI in drug discovery. Efforts must be made to ensure that AI models are trained on diverse and representative data sets and that their predictions are validated across different populations and settings. Additionally, robust data protection measures must be implemented to safeguard patient data and maintain public trust in AI-powered drug discovery.

Despite these challenges, the potential benefits of AI-powered drug discovery for neglected diseases are substantial. By accelerating the identification and development of new treatments, AI can significantly reduce the burden of neglected diseases in the developing world, improving health outcomes and enhancing the quality of life for millions of people. The scalability and efficiency of AI-driven drug discovery could lead to more cost-effective treatment options, making it possible to address neglected diseases even in resource-limited settings. This could have a transformative impact on global health equity, helping to close the gap between high- and low-income countries regarding access to essential medicines.

Furthermore, the successful application of AI in drug discovery for neglected diseases could serve as a model for other areas of global health. For example, using AI to identify new treatments for infectious diseases such as coronavirus disease 2019 has already shown significant promise, demonstrating the potential of AI to address a wide range of public health challenges. By acknowledging both the potential and limitations of AI and emphasising the importance of human oversight, researchers can continue to leverage AI to address other pressing global health issues, ultimately improving health outcomes and advancing health equity.

## CONCLUSION

AI-powered drug discovery offers a powerful tool for addressing the global burden of neglected diseases, particularly in the developing world. By accelerating the identification and development of new drugs, it has the potential to transform public health strategies and bring much-needed treatments to market more quickly and cost-effectively. Concrete actions are needed, however, to fully harness this potential. Policymakers must create supportive regulatory frameworks that encourage the ethical use of AI in health care, while also investing in the necessary technological infrastructure to support AI research and implementation in low-resource settings. Furthermore, fostering international collaborations among governments, NGOs, and academic institutions will be key to sharing knowledge, resources, and expertise, ultimately accelerating drug discovery efforts for neglected diseases. Yet first and foremost, the successful implementation of AI in drug discovery requires careful attention to ethical considerations, capacity building in low-resource settings, and a commitment to ensuring that the benefits of this technology are equitably distributed. As research in this field progresses, AI-powered drug discovery is poised to play an increasingly important role in improving global health outcomes and advancing health equity.
